# Retraction: Integration of Neuroscience and Entrepreneurship: A Systematic Review and Bibliometric Analysis

**DOI:** 10.3389/fpsyg.2024.1490714

**Published:** 2024-09-12

**Authors:** Frontiers Editorial Office

The journal retracts the 01 April 2022 article cited above.

Following publication, concerns were raised regarding the scientific validity of the article. An investigation was conducted in accordance with Frontiers' policies. It was found that the complaints were valid and that the article does not meet the standards of editorial and scientific soundness for Frontiers in Psychology; therefore, the article has been retracted.

The retraction was approved by the Chief Editors for Frontiers in Psychology and the Chief Executive Editor of Frontiers. The authors do not approve of the retraction.

